# Inhibition of peptide aggregation by means of enzymatic phosphorylation

**DOI:** 10.3762/bjoc.12.240

**Published:** 2016-11-18

**Authors:** Kristin Folmert, Malgorzata Broncel, Hans v. Berlepsch, Christopher Hans Ullrich, Mary-Ann Siegert, Beate Koksch

**Affiliations:** 1 Department of Chemistry and Biochemistry, Freie Universität Berlin, Takustr. 3, 14195 Berlin, Germany; 2 The Institute of Cancer Research, 237 Fulham Road, London SW3 6JB, UK; 3 Deutsche Bahn, Umweltservice, Bahntechnikerring 74, 14774 Kirchmöser, Germany; 4 Department of Organic Chemistry, Technische Universität Berlin, Strasse des 17. Juni 124, 10623 Berlin, Germany

**Keywords:** aggregation, amyloids, peptide models, peptide phosphorylation, protein folding

## Abstract

As is the case in numerous natural processes, enzymatic phosphorylation can be used in the laboratory to influence the conformational populations of proteins. In nature, this information is used for signal transduction or energy transfer, but has also been shown to play an important role in many diseases like tauopathies or diabetes. With the goal of determining the effect of phosphorylation on amyloid fibril formation, we designed a model peptide which combines structural characteristics of α-helical coiled-coils and β-sheets in one sequence. This peptide undergoes a conformational transition from soluble structures into insoluble amyloid fibrils over time and under physiological conditions and contains a recognition motif for PKA (cAMP-dependent protein kinase) that enables enzymatic phosphorylation. We have analyzed the pathway of amyloid formation and the influence of enzymatic phosphorylation on the different states along the conformational transition from random-coil to β-sheet-rich oligomers to protofilaments and on to insoluble amyloid fibrils, and we found a remarkable directing effect from β-sheet-rich structures to unfolded structures in the initial growth phase, in which small oligomers and protofilaments prevail if the peptide is phosphorylated.

## Introduction

Amyloid fibrils are one of the most important and studied self-assembled materials in nature. A wide range of peptides and proteins with various primary sequences and functions are able to form these wel organized and highly stable aggregates under multiple conditions [1]. The morphology ranges from liquid crystals to ribbons, rigid nanotubes and funnels [2]. Although amyloid aggregates are mostly known for pathologic events like Alzheimer’s and Parkinson’s disease or type two diabetes [3–6], they also combine the potential for applications with biological function with desirable mechanical properties. Many such peptide aggregates display exceptional stability, mechanical strength, stability at high temperatures and they are resistant towards enzymatic degradation [2]. Several studies have demonstrated the utilization of amyloids as functional templates like conductive nanowires [7], water-filled nanotubes [8] or biocompatible hydrogels [9]. Likewise, nature uses amyloid-like cross-β-sheet-rich conformations to store peptide hormones in secretory granules of the endocrine system [10]. As another example it was found that mammalian Pmel17 amyloid templates and accelerates the covalent polymerization of reactive small molecules into melanin, an important biopolymer that protects against a broad range of cytotoxic insults, including UV and oxidative damage [11]. While amyloid morphologies and events which induce amyloid formation are the subject of numerous studies, the pathway of aggregation from soluble α-helical-rich or partially unfolded peptides into insoluble β-sheet-rich amyloid fibrils remains unclear. Several studies discovered parameters of peptide environment, like pH [12–13], oxidative stress [14], presence of organic components [15–16] or metal ions [17–18], to have a crucial influence on the conformational transition. The challenging physicochemical properties, the low solubility and the tendency to aggregate make natural amyloid-forming peptides difficult to synthesize and complicates detailed structural characterization. One possibility to overcome these problems is the use of de novo designed model peptides [19]. The identification of short domains in peptide sequence which make a peptide prone to amyloid formation [20–23] inspired the design of a series of amyloid-forming model peptides with defined characteristics, outstanding stability, regular fibrous architecture and high synthetic accessibility with numerous chemoselective ligation and modification methods [24–26]. Also, various post-translational modifications, like phosphorylation or glycosylation have been studied as aggregation triggers [27–29]. Phosphorylation caused by abnormal activities of phosphatases and/or kinases is associated with known diseases such as cancer [30], multiple sclerosis [31], diabetes [32] and Alzheimer’s disease [33]. The accumulation of hyperphosphorylated tau protein as trigger for several dementias was intensively discussed [33–35]. Moreover, the effect of enzymatic phosphorylation on Alzheimer-relevant APP was demonstrated already decades ago [36] and has been followed up in plenty of studies since then [37–40]. Phosphorylation experiments were also transferred to model peptide systems by our group. We explored a 26-residue coiled-coil peptide which undergoes a conformational transition to amyloid fibrils in 24 hours under physiological conditions [41], but remains random coil if one of three serine residues carries a phosphate group [27]. The aggregation process could be restored by addition of Lambda Protein Phosphatase that removes the phosphate group [42]. It is widely accepted that this reversible process of phosphorylation directs diverse properties of peptides or proteins in nature, ranging from interactions with other proteins and nucleic acids to subcellular localization and binding [43]. Additionally, phosphorylation may be found to direct desired behaviors in the currently intensively studied area of amyloid-based biomaterials. In this report we show the use of the negatively charged phosphate group to control the aggregation process. Using PKA and ATP (adenosine triphosphate) as phosphate group donor, we studied the impact of the phosphate group on the oligomerization pathway at different conformational states from random coil, over β-sheet monomers to protofilaments and amyloid fibrils. Furthermore, we demonstrate the importance of the influence of phosphorylation components on the peptide conformation during this process. This knowledge could become a useful tool in employing enzymatic phosphorylation and dephosphorylation as triggers for or inhibitors of amyloid formation, as it was previously shown for a self-assembling, supramolecular hydrogel [44].

## Results

### Peptide model

**Scheme 1:**
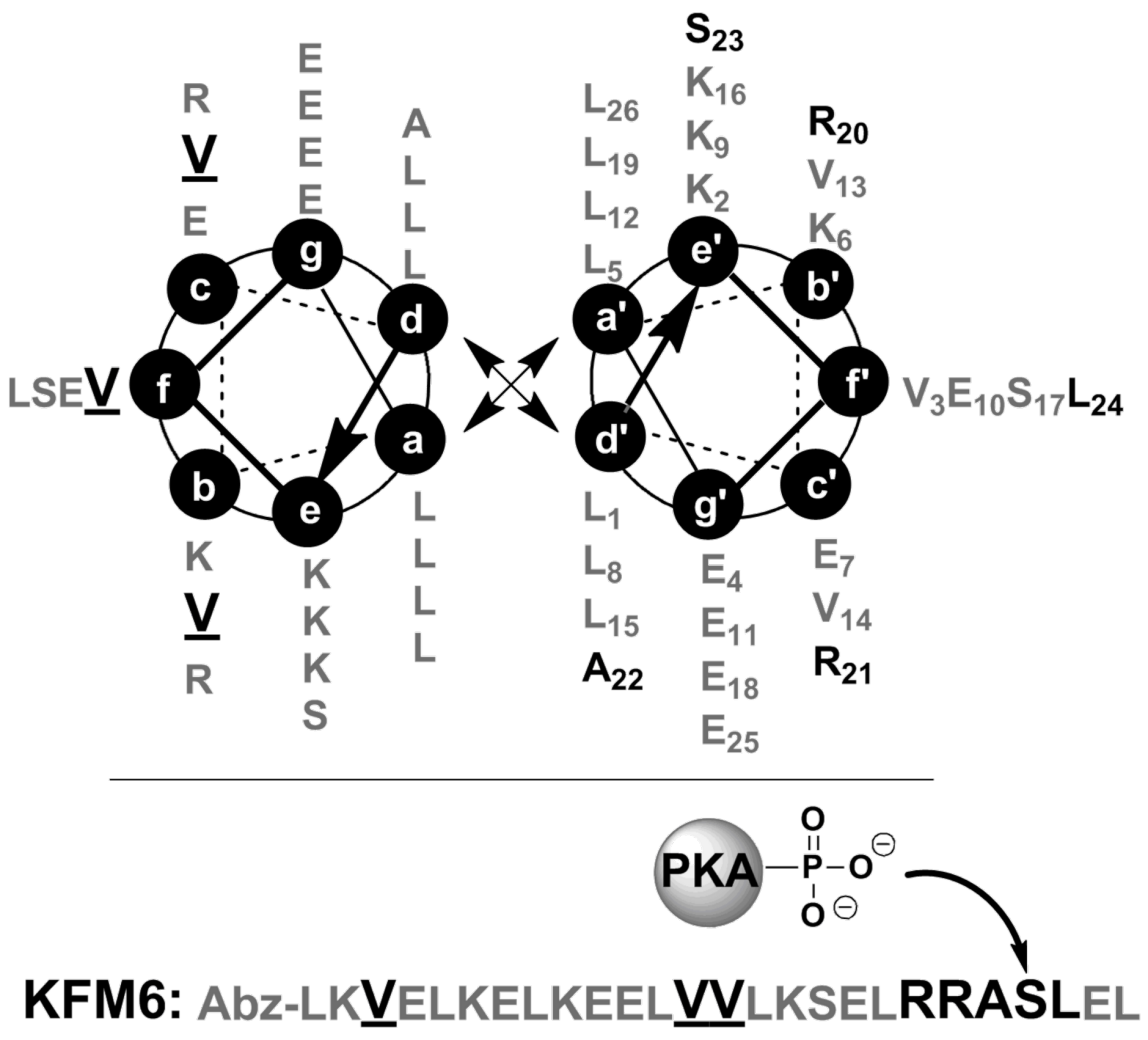
Helical wheel projection of two parallel helical strands and primary structure of KFM6. The recognition motif KFM6(20–24) for PKA is given in bold and valine residues are bold and underlined.

Peptide KFM6 follows a typical coiled-coil heptad repeat sequence and it includes five amino acids (-R-R-A-S-L-) in positions 20–24, in proximity to the C-terminus, that serve as the recognition motif for PKA. The crucial role of this recognition motif for efficient phosphorylation has long been established [45]. It is also known that enzyme activity depends upon the local flexibility and solvent-exposed position of the target amino acid in the peptide [46]. Scheme 1 depicts the key design features, which are based on one of our previously published peptides [47], and the primary structure of KFM6. The nonpolar leucine residues at positions a and d contribute to thermal stability by hydrophobic core packing of the leucine zipper motif (“knobs-into-holes principle”). Charged amino acids at positions e, g, b, and c stabilize the coiled-coil by intramolecular electrostatic attractions between glutamates and lysines.

Intermolecular coulombic interactions between e and g direct the monomers into a parallel dimeric orientation. The solvent-exposed position f is occupied by serine for better solubility. One site of each position b, c, and f contains valine, making the system prone to amyloid formation. To ensure the UV-activity of the peptide as analytical tool, Abz (anthranilic acid) was coupled to the N-terminus. The resulting peptide contains elements of α-helices and β-sheets, and a recognition site for PKA. To investigate the structural changes that KFM6 undergoes in the absence of phosphorylation, time-dependent CD (circular dichroism) measurements were performed. To ensure comparable and reproducible starting conditions, a disaggregation and concentration validation assay using HFIP (1,1,1,3,3,3-hexafluoropropan-2-ol) was performed. Thus, all aggregates that may have formed during peptide synthesis and purification were disrupted.

**Figure 1:**
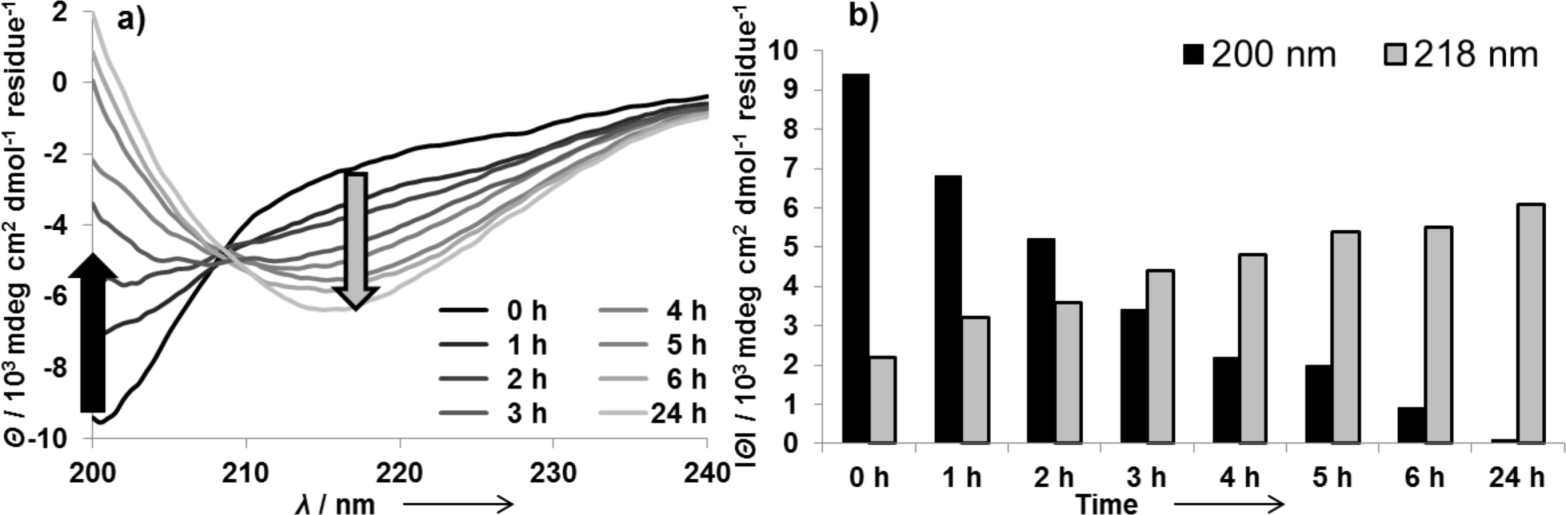
a) Time-dependent CD spectra and b) corresponding CD-minimum plot for random coil (black) and β-sheet (grey) of 15 µM KFM6 in 50 mM Tris/HCl buffer with 10 mM MgCl_2_ at pH 7.5 and 24 °C.

As shown in Figure 1 the peptide KFM6 adopts a random structure upon dissolution in buffer and undergoes a time-resolved conformational transition to β-sheet-rich amyloid fibrils within 24 hours. The CD spectra (Figure 1a) show a typical minimum at 200 nm indicating a random-coil conformation with decreasing intensity over 24 hours, and another increasing minimum at 218 nm, which is characteristic for β-sheets [48]. Absolute values of ellipticity IΘI for 200 or 218 nm are plotted versus the time (Figure 1b) for better visualization.

**Figure 2:**
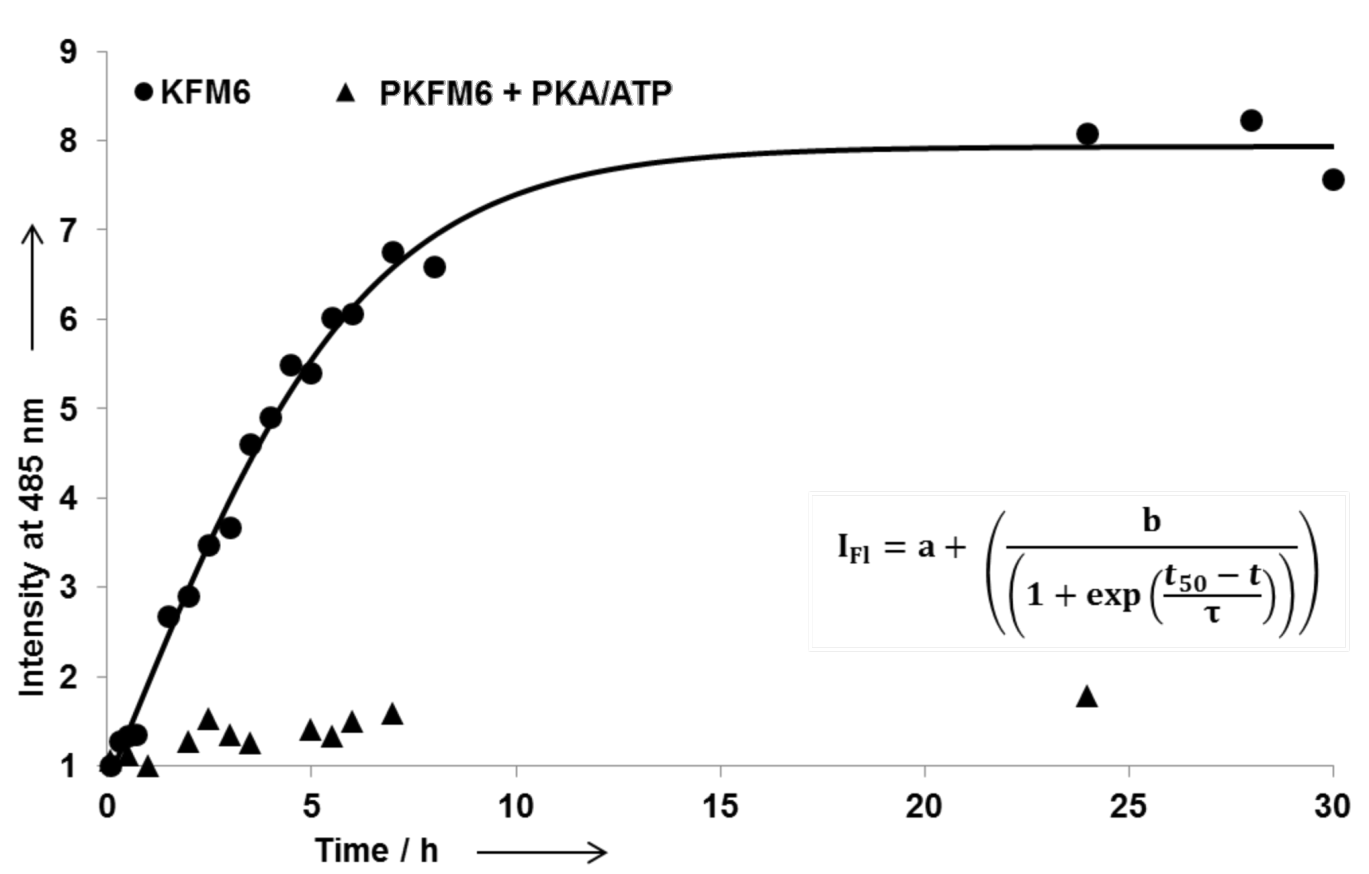
ThT-binding assay of of 15 µM KFM6 in 50 mM Tris/HCl buffer with 10 mM MgCl_2_ at pH 7.5, 24 °C, and a ThT concentration of 20 µM. The amyloid growth process was monitored for untreated peptide (circles) and peptide in the presence of PKA and ATP (triangles). All values were determined in triplicate and are normalized based on the initial fluorescence intensity (*t* = 5 min; λ = 485 nm).

Although a white precipitate indicates the formation of aggregates, amyloid fibril formation was studied by means of a ThT (thioflavine T) binding assay (Figure 2). ThT yields in an enhanced fluorescence signal at 485 nm when bound to amyloid fibrils, and can be used to determine the kinetics of fibril formation [49]. KFM6 shows a strongly increasing fluorescence signal without lag time, reaching a plateau after ten hours (Figure 2, circles).

**Figure 3:**
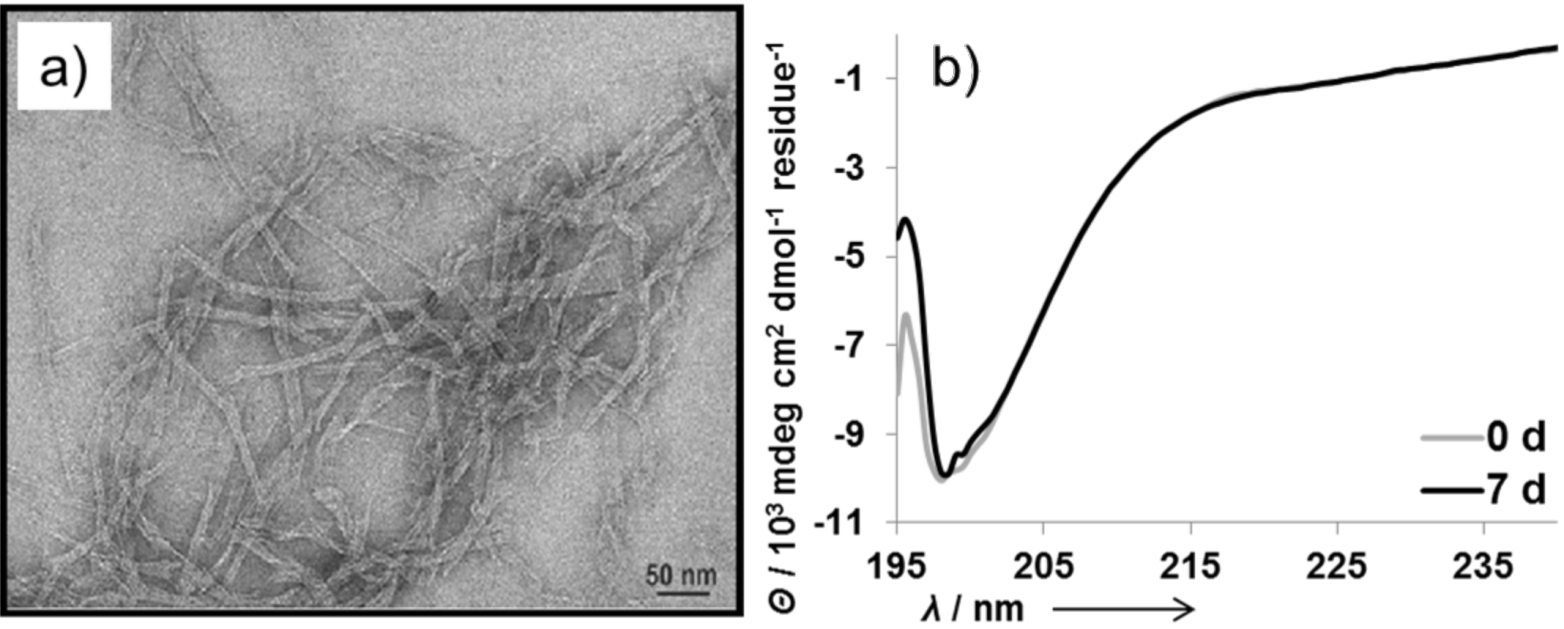
a) TEM micrograph of 15 µM KFM6 in 50 mM Tris/HCl buffer with 10 mM MgCl_2_ at pH 7.5 and 24 °C. The peptide was incubated for 24 h in a closed cuvette in the presence of 5000 U PKA and 200 µM ATP and stained with 1% PTA (phosphortungstic acid). b) Time-dependent CD spectra of 30 µM P_C_KFM6 in 50 mM Tris/HCl buffer with 10 mM MgCl_2_ pH 7.5 and 24 °C.

The displayed data indicate a fast amyloid growth process that appears to begin upon dissolution in buffer. This observation is in good agreement with the CD spectra, in which no lag time was observed. To discover the morphology of the amyloid fibrils, a TEM (transmission electron micrograph) was obtained at 24 hours of incubation (Figure 3a). The TEM shows typical amyloid morphologies such as extended tubular fibers, some of them ending in funnels. The protofilaments are frequently well resolved. Their spacing is about 3.5 nm, which is in good agreement with estimates for the protofilaments of other related peptides [47].

### Enzymatic phosphorylation

To investigate the influence of enzymatic phosphorylation on the aggregation process, buffer containing PKA and ATP was prepared and used to dissolve lyophilized KFM6. PKA is the most studied and structurally well-characterized protein kinase, and it is known to have relatively broad substrate specificity due to its roles in the regulation of energy metabolism, growth, signal transduction, and apoptosis of cells [50]. The serine/threonine kinase PKA uses ATP as phosphate donor. It has to be activated from its natural species with cAMP which leads to dissociation of PKA in two regular and two catalytic subunits [51]. We used the preactivated catalytic subunit of PKA to perform the reactions. The structural behavior of KFM6 during enzymatic phosphorylation was determined by different analytical methods.

**Figure 4:**
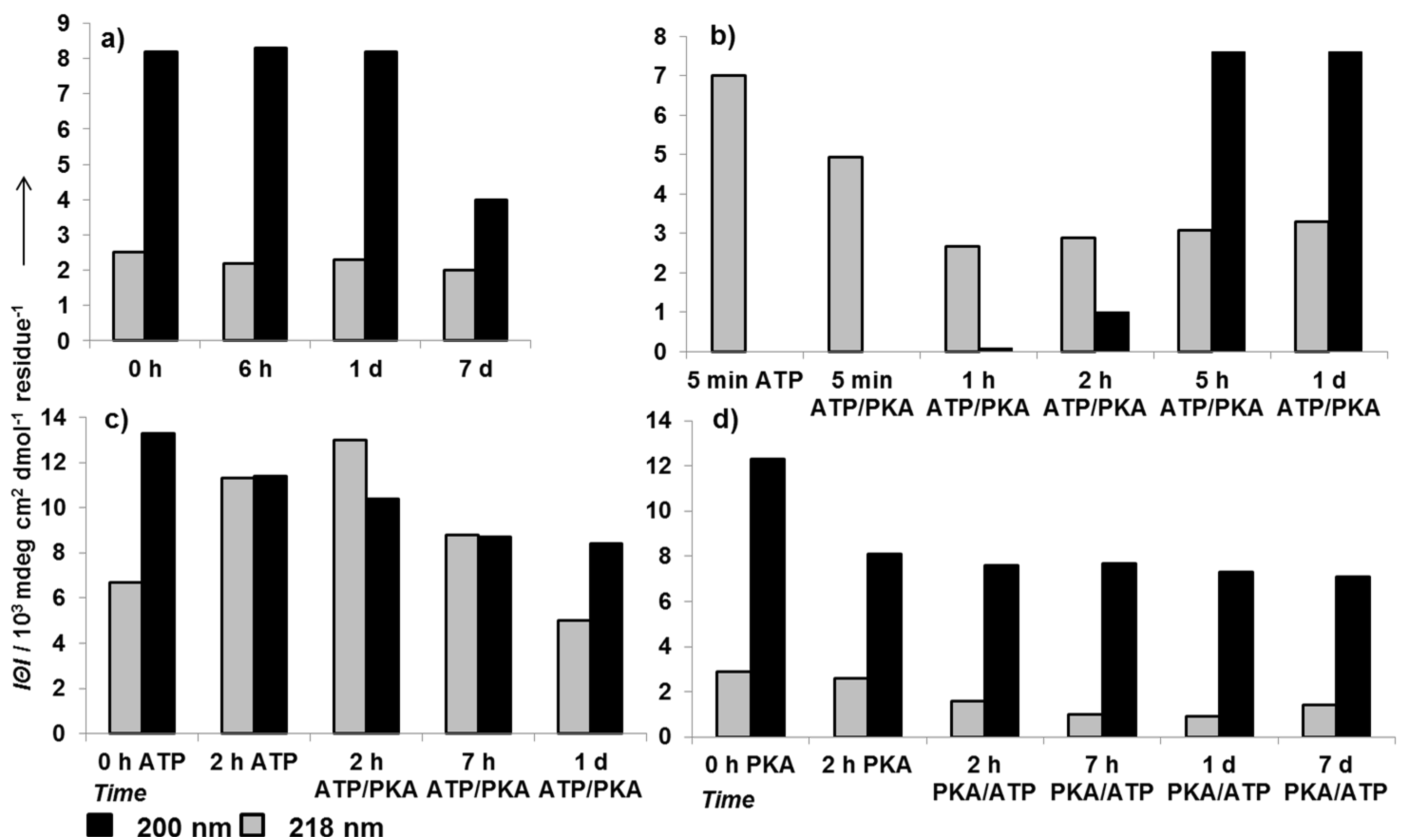
a) Enzymatic phosphorylation of 15 µM KFM6 in time-dependent CD-minimum plots. Addition of both 200 µM ATP and 5000 U PKA at time of dissolution of peptide; b) ATP-induced β-sheet formation, reversed by the addition of PKA after five minutes of incubation; c) the influence of ATP during the first two hours of conformational change and effect of two hours delayed phosphorylation; and d) the inverse experiment with PKA.

The ThT binding assay shows no increase in amyloid formation compared to the unphosphorylated sample over 24 hours (Figure 2, triangles) and the CD-minimum plot indicates a large population of random structures and a constantly low intensity for β-sheets over seven days of incubation (Figure 4a). Together with the TEM micrograph, which shows no sign of amyloid morphologies or other higher oligomers, the results suggest the inhibition of amyloid formation of KMF6 by enzymatic phosphorylation. Also, no precipitation was observed during all enzymatic phosphorylation experiments, which is a further indicator for less aggregate formation.

To learn more about the influence of phosphorylation components on the pathway of aggregation and at which state the growth of oligomers is stopped by phosphorylation, we performed time-dependent enzymatic phosphorylation studies. Therefore, ATP and PKA were added separately at time zero and the other one was added after a certain time assuming that the phosphorylation of KFM6 begins immediately when both phosphorylation components are present in solution. In this way, the influence of ATP or PKA on amyloid fibril formation was monitored as control. In Figure 4b, the persistent minimum at 218 nm for β-sheets and the apparent absence of a random-coil structure suggest that ATP accelerates the aggregation process, which is in a good agreement with previous studies in which organic anions were found to enhance the formation of amyloid fibrils [16]. When, after a five minute incubation time of KFM6 with ATP, PKA is added, the concentration of β-sheets decreases and the minimum at 200 nm for random structures strongly increases (Figure 4b). This conformational transition shows a strong directing effect of enzymatic phosphorylation in the early stages of aggregate growth, during which small oligomers and protofilaments prevail. If the peptide was incubated with PKA for two hours (Figure 4d), the soluble peptide concentration decreases, what could be an effect of aggregation. After two hours and induced phosphorylation, the peptide concentration and structure seems to be stabilized over at least seven days. The inverse experiment, with ATP incubation for two hours, results in an intensive increase of β-sheet structures in KFM6 (Figure 4c). After induced phosphorylation, the concentration of β-sheets decreased in good agreement with the previous observation. To further clarify the conformational shift the 200/218 nm ratio of some CD experiments was depicted in Supporting Information File 1 (Figure S3).

### Chemical phosphorylation

To demonstrate the directing effect of the phosphate group towards soluble random structures, chemically phosphorylated P_C_KFM6 was synthesized for control experiments. The peptide was dissolved in HFIP stock solution, as was unphosphorylated KFM6. For CD experiments P_C_KFM6 was prepared in two-fold higher concentration (30 µM) compared to KFM6, with the intention to make it more prone to aggregation. However, only random structures were observed after seven days (Figure 3b). A TEM was taken 24 hours after dissolving and incubating the peptide. P_C_KFM6 shows no morphologies of amyloid fibrils, its preliminary states or other higher order oligomers. Also no precipitation was observed. These results indicate high stability of the chemically phosphorylated peptide in comparison to the unphosphorylated analog.

## Discussion

In the accepted model pathway of amyloid formation, a lag phase populated by functional soluble peptide structures is followed by partial unfolding, then a conformational transition into β-sheet monomers follows and these assemble into oligomers and protofilaments which then form amyloid fibrils [26,52]. While most inhibition studies concentrate on the later phase of this process, the results we present here suggest a strong directing effect to random structures during the initial phase of the aggregation process concomitant with enzymatic phosphorylation. Phosphorylation experiments at different time points of the aggregation process suggest a time-dependent inhibitory effect, which becomes less efficient with increasing concentration of amyloid fibrils. The impact of phosphorylation must be considered with respect to three properties: steric bulk, charge and hydrophobicity. It was calculated that phosphorylation increases the van der Waals volume of the serine side chain by a factor of two [27] and it seems natural that flexible structures and solvent exposed positions should be preferred for both the interaction of the recognition site with the enzyme and the resulting, bulky phosphate group. Nevertheless, comparative studies between phosphate and β-galactose, incorporated in an amyloid forming coiled-coil peptide, showed no significant structural influence of the three times larger sugar residue, whereas the phosphate group directed the peptide into unfolded structures [27]. Additionally, the phosphate group introduces a formal charge of −2 to the peptide at pH 7.5. This leads to intramolecular Coulombic repulsions between negatively charged glutamate residues and the phosphate, or in electrostatic pairing with positively charged lysine or arginine residues, resulting in the perturbation of higher ordered secondary structures [53]. The impact of electrostatics on peptide and protein folding and self-assembly has been studied extensively [12,53], and it is known that the impact of the phosphate group charge strongly depends on the pH and the neighboring residues in the peptide [53–54]. In contrast to the accepted model where negatively charged phosphates must be close to the N-terminus to have a stabilizing effect on the original secondary structure [55], we demonstrate that phosphorylation proximal to the C-terminus can also significantly inhibit amyloid formation.

Delayed ATP or PKA addition lead in all cases to a stop of the amyloid formation and to stabilization of remaining soluble structures, as no further precipitation was observed. It remains unclear whether the decreasing effect of the phosphorylation during later stages of the aggregation is due to the lower flexibility of the recognition site, leading to slow reaction kinetics of the enzyme or to the competitive effects of nucleation-induced aggregation and phosphorylation. ^31^P NMR experiments displaying the enzymatic phosphorylation over 12 hours with measurements every 30 minutes suggest rapid phosphorylation, as no change in the integral ratio of β-phosphate to α-phosphate of ATP was observed subsequent to the first time point (Supporting Information File 1, Figure S4). These observations support the theory that fully formed aggregates in a protofilamental or amyloidal stage cannot be accessed by the enzyme. This could be either a consequence of the low solubility or the tight cross-β-structure of the amyloid fibrils [56]. Furthermore, the presence of amyloid fibrils is known to have a strong promoting effect on the aggregation process [57–58], whereby nucleation, together with the unfavorable structure could conceal the effect of phosphorylation. Thus, a further proof was the chemically phosphorylated peptide P_C_KFM6 which completely lost its ability to undergo a conformational transition and remains random coil even at higher concentrations.

The conformational transition to the β-sheet-rich structures is provided by the incorporation of hydrophobic domains, which can also decrease the overall solubility of the peptide. Calculated secondary structure propensities, using the TANGO algorithm, identify a nine amino acid long β-aggregation domain (E-L-V-V-L-K-S-E-L-) for KFM6 (Supporting Information File 1, Figure S5). Those aggregation domains are found in almost every peptide with the propensity to form amyloids [59–60]. The recognition site for PKA is located next to the aggregation domain of KFM6, with just three amino acids distance to the phosphorylated serine residue. The short distance to this hydrophobic aggregation domain could enable an interaction with the polar phosphate group, resulting in the suppression of the β-aggregation propensity. This is in accordance with simulations carried out by Rousseau et al. who proposed that charged amino acid residues flanking aggregating peptide segments could act as gatekeeper residues that reduce the aggregation propensity of the peptide [61–63]. Nevertheless, the change of the serine in position 23 of KFM6 to a glutamic acid does not influence the calculated propensities to form β-aggregates as the phosphorylation does (Supporting Information File 1, Figure S5). It has also been reported that not only the charge, but also the position of the phosphate group can influence the amyloid formation and even the morphology of the formed fibrils [54,64]. Determining the relative contributions of the above factors to the role of phosphorylation in the amyloid forming pathway is a major challenge, however, crucial for the molecular understanding of the process.

## Conclusion

Post-translational modifications like phosphorylation have become the most promising approach to examine and control the pathway of amyloid fibrillization. For a better understanding of the challenges of phosphorylation in this context, we designed a model peptide which undergoes a conformational transition from soluble structures to amyloids under physiological conditions and additionally presents a recognition site for PKA. During the initial growth phase, in which small oligomers and protofilaments are assumed to prevail, our results demonstrate a remarkable directing effect towards unfolded structures, if the peptide was phosphorylated close to the hydrophobic aggregation domain of the peptide. However, the enzymatic phosphorylation had to be realised on flexible secondary structures and couldn’t dissipate grown aggregates in our experiments. The ability to identify those influences is a major challenge to use enzymatic phosphorylation as a tool to control the aggregation process and for the development of new functional biomaterials based on amyloid morphologies.

## Experimental

### Peptide synthesis, purification and characterization

Peptides were synthesized manually according to standard Fmoc chemistry using preloaded Fmoc-Leu-NovaSyn^®^TGA resin (0.3 mmol g^−1^, Novabiochem). Standard couplings were performed in DMF with Fmoc-amino acids and TBTU (O-(benzotriazole-1-yl)-*N*,*N*,*N*’,*N*’-tetramethyluronium tetrafluoroborate)/HOBt (1-hydroxybenzotriazole)/DIC (*N*,*N*’-diisopropylcarbodiimide) in eight-fold and NaClO_4_ in ten-fold excess with double couplings of one hour coupling time. Fmoc-Ser(PO(OBzl)OH)-OH was activated with HATU (O-(7-azabenzotriazol-1-yl)-*N*,*N*,*N*’,*N*’-tetramethyluronium hexafluorophosphate)/HOBt five-fold and fifteen-fold excess of DIPEA (*N*,*N*-diisopropylethylamine) with respect to the resin and two hour double couplings. A mixture of DBU (1,8-diazabicyclo[5,4,0]undec-7-ene) and piperidine (2% each) in DMF was used for Fmoc-deprotection (3 × 10 min). The resin was washed between each step with DMF and DCM (3 × 6 mL each). Peptides were cleaved from the resin by treatment with 2 mL TFA (trifluoroacetic acid/iPr_3_SiH/H_2_O (90:9:1)) for three hours. The resin was washed twice with 1 mL TFA and DCM, and excess of solvent was removed by evaporation. The peptides were precipitated with cold Et_2_O, pelleted by centrifugation and dried by lyophilization. All peptides were purified with preparative reversed-phase HPLC by using a Knauer Smartline system (Knauer, Berlin, Germany) equipped with a Luna C8 column (10 u, 250 × 21.20 mm, Phenomenex, Torrance, CA, USA) running with a ACN/ H_2_O + 0.1% TFA gradient at a flow rate of 20 mL min^−1^ flow. The crude peptide was dissolved in 5 mL ACN/H_2_O/DMSO (1:1:1). The collected fractions were evaporated and the purified peptide was dissolved in 2 mL of water and lyophilized to give the peptide as a white powder. Pure peptides were characterized by means of analytical HPLC and ESI–ToF mass spectrometry. Analytical HPLC was carried out with a VWR-Hitachi Elite LaCrome system (VWR, Darmstadt, Germany) using a Luna C8 column (5 u, 250 × 4.6 mm, Phenomenex, Torrance, CA, USA). The mass to charge ratios were determined with an Agilent 6210 ESI–ToF (Agilent Technologies, Santa Clara, CA, USA). Peptide solutions were injected by a syringe pump with a flow rate of 20 µL min^−1^. Spray voltage was set to 4000 V, drying gas flow rate was 5 L min^−1^ and gas temperature was set to 300 °C. Both retention times and peptide masses are given in Supporting Information File 1 (Table S1).

#### Concentration determination

A stock solution was prepared by dissolving the purified, Abz labeled peptide in HFIP (≈1 mg mL^−1^) and sonicating for 15 minutes to dissolve all aggregates. 50 µL of this solution were aliquoted and dried under nitrogen flow, before the residue was dissolved in 1 mL 50 mM Tris/HCl buffer containing 10 mM MgCl_2_ at pH 7.5. UV spectra were recorded in a 1 cm path length cuvette using a Cary 50 UV–vis spectrophotometer (Varian, Palo Alto, CA, USA) and the absorbance maximum at 312 nm was compared to a standard curve of the dipeptide H_2_N-Abz-Gly-OH·HCl to calculate the concentration of the peptide stock solution. The stock solution was stored at −20 °C.

#### Circular dichroism

CD spectra were recorded by using a Jasco J-810 spectropolarimeter (Jasco, Gross-Umstadt, Germany) at 24 °C (Jasco PTC-348W1 peltier thermostat) using 0.2 mm path length Quartz Suprasil cuvettes (Hellma, Müllheim, Germany). After background correction, the spectra were averaged over three scans (λ = 195–240 nm; 0.5 nm intervals; 2 mm bandwidth; 4 s response time, 100 nm min^−1^ scanning speed). Ellipticity was normalized to concentration (*c* [mol L^−1^]), number of residues (*n* = 27, including the N-terminal Abz group) and path length (l [cm]) by using Equation 1 in which Θ_obs_ is the measured ellipticity in millidegrees and Θ is the mean residue ellipticity in 10^3^ mdeg cm^2^ dmol^−1^ residue^−1^.

[1]
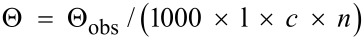


Aliquots of the peptide stock solution were dried under nitrogen flow and, immediately before measurement, dissolved in 350 µL 50 mM Tris/HCl buffer with 10 mM MgCl_2_, including 5000 U PKA and 200 µM ATP for the enzymatic phosphorylation studies. The pH was adjusted to 7.5 with 1 M NaOH. For time-dependent phosphorylation experiments, the buffer was prepared with just one of the phosphorylation components, while the other one was added at different time points.

#### Thioflavin T fluorescence spectroscopy

Fluorescence spectra were recorded by using a 1 cm path length quartz cuvette (Hellma, Müllheim, Germany) and a luminescence spectrometer LS50B (Perkin-Elmer, Boston, MA, USA). Spectra were recorded at room temperature from 470−500 nm after excitation at 450 nm (excitation slit width 5 nm; emission slit width 20 nm; scan speed = 500 nm min^−1^; accumulations = 5). Sample preparation was the same as for CD with a volume of 500 µL buffer including 20 µM ThT. The fluorescence intensity at 485 nm was recorded at different time points over a total time period of 24 hours and normalized to the starting value at *t* = 5 min of one. The shown plots represent an average of three independent measurements.

#### Transmission electron microscopy

Peptides, prepared as for CD spectroscopy measurements, were examined after 24 hours. Aliquots (6 µL) of the corresponding solution were placed for 60 seconds onto glow-discharged (60 s plasma treatment at 8 W in BAL-TEC MED 020), carbon-coated collodium films on 400-mesh copper grids (Leica Microsystems, Wetzlar, Germany). After blotting and negative staining with 1% PTA, the grids were left to air-dry. The TEM images were recorded with a Philips CM12 transmission electron microscope (FEI, Oregon, USA) at 100 kV acceleration voltage and at a primary magnification of 58000× on Kodak SO-163 negative film by using a defocus of 900 nm.

## Supporting Information

File 1 Description of further methods and additional figures.
