# Supplementary material for: Inhibition of peptide aggregation by means of enzymatic phosphorylation
Source: Beilstein J Org Chem. 2016 Nov 18;12:2462–70. doi: 10.3762/bjoc.12.240 (PMC5238555; doi:10.3762/bjoc.12.240)
Supplement: File 1 — Description of further methods and additional figures. [file Beilstein_J_Org_Chem-12-2462-s001.pdf]

Supporting Information

for

Inhibition of peptide aggregation by means of enzymatic phosphorylation

Kristin Folmert[1], Malgorzata Broncel[2], Hans v. Berlepsch[1], Christopher H. Ullrich[3], Mary-Ann Siegert[4] and Beate Koksch[1]*

Address: [1]Department of Chemistry and Biochemistry, Freie Universität Berlin, Takustr. 3, 14195 Berlin, Germany, [2]The Institute of Cancer Research, 237 Fulham Road, London SW3 6JB, UK, [3]Deutsche Bahn, Umweltservice, Bahntechnikerring 74, 14774 Kirchmöser, Germany and [4]Department of Organic Chemistry, Technische Universität Berlin, Strasse des 17. Juni 124, 10623 Berlin, Germany

Email: Beate Koksch - Beate.Koksch@fu-berlin.de

* Corresponding author

Description of further methods and additional figures

1. Materials

Fmoc-L-amino acids were purchased from ORPEGEN Peptide Chemicals GmbH (Heidelberg, Germany). Fmoc-Ser(PO(OBzl)OH)-OH and Boc-Abz-OH were obtained from Bachem (Weil am Rhein, Germany). Fmoc-Leu Nova Syn®TGA-resin with 0.3 mmol/g loading was from the company NovaBiochem (Wolfshagen, Germany). PKA and ATP were purchased from New England BioLabs (Frankfurt am Main, Germany). All solvents and common chemicals were used from Acros Organics (Geel, Germany) without further purification.

2. Peptide characterization

ESI-ToF

Mass-to-charge ratios were determined with an Agilent 6210 ESI-ToF (Agilent Technologies, Santa Clara, CA, USA). Peptides were dissolved in ACN/H_2O (1:1) and the peptide solutions were injected by a syringe pump with a flow rate of 10 µL min^{-1}. Spray voltage was set to 4000 V, drying gas flow rate was 5 L min^{-1} and gas temperature was set to 300 °C.

HPLC

Analytical HPLC was carried out with a VWR-Hitachi Elite LaCrome system (VWR, Darmstadt, Germany) and a Luna C8 column (5 u, 250 × 4.6 mm, Phenomenex, Torrance, CA, USA) was used for data analysis. LaChrom-software (Version 3.0, Merck) was used to analyze data. The gradient was a mixture of H_2O and ACN with 0.1% TFA from 5 to 70% ACN over 30 minutes followed by a washing and calibration time. Peptides were detected at a wavelength of 220 nm. Phosphorylated peptide PcKFM6 is degrading under HPLC conditions. The products of degradation, KFM6 and polyphosphoric acid, appear with the same retention time.

Table S1: Determined and calculated mass to charge ratios and retention times on analytical HPLC of the peptides.

peptide	charge	calc. mass (*m*/*z*)	det. mass (*m*/*z*)	t_R (min)
KFM6	0	3980.9592	–	23.48
	+2	1591.9174	1592.4449	
	+3	1061.6142	1061.9654	
	+4	796.4626	796.7276	
P_CKFM6	0	4077.9292	–	27.15
	+2	1640.9174	1640.9378	
	+3	1094.2808	1094.4539	
	+4	820.9626	820.9653	

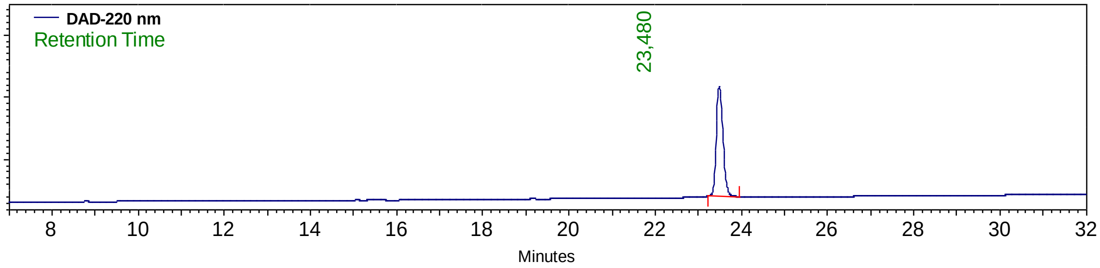

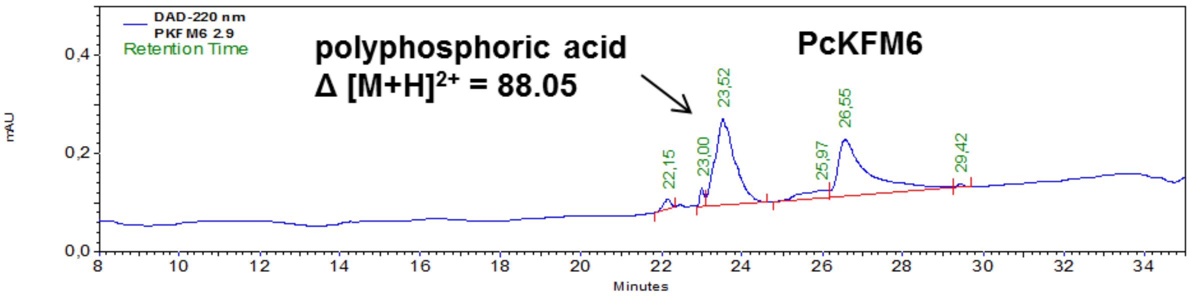

Figure S1: HPLC chromatogram of KFM6 (top) and PcKFM6 (below).

3. CD spectroscopic analysis

Time-dependent CD spectra of 15 µM KFM6 to which both 200 µM ATP and 5000 U PKA were added. CD spectra were recorded by using a Jasco J-810 spectropolarimeter (Jasco, Gross-Umstadt, Germany) at 24 °C (Jasco PTC-348W1 peltier thermostat) using 0.2 mm path length Quartz Suprasil cuvettes (Hellma, Müllheim, Germany). After background correction, the spectra were averaged over three scans (λ = 195–240 nm; 0.5 nm intervals; 2 mm bandwidth; 4 s response time, 100 nm min^{-1} scanning speed).

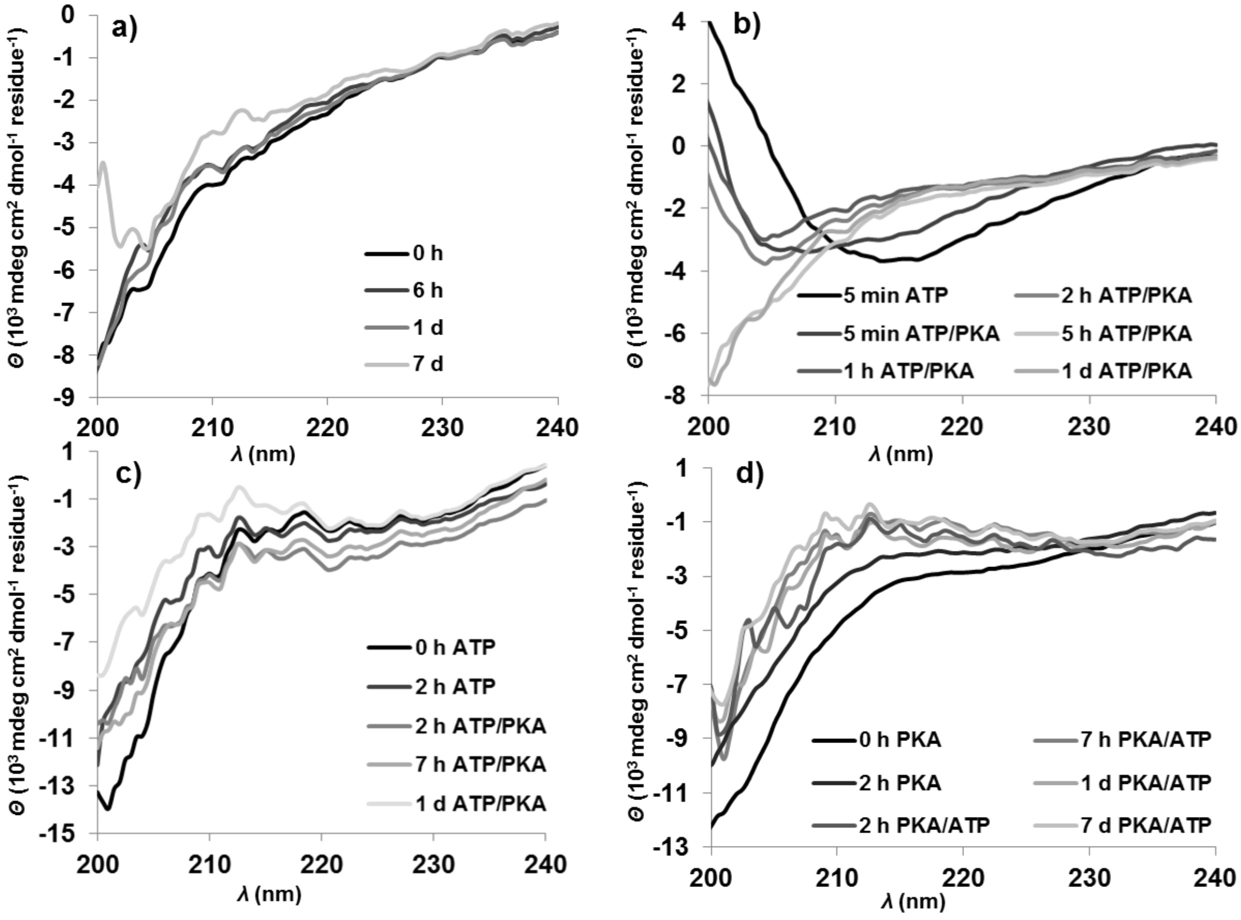

Figure S2: CD spectra of enzymatic phosphorylation of 15 µM KFM6 in time-dependent CD-minimum plots. Addition of both 200 µM ATP and 5000 U PKA at time of dissolution of peptide (a); ATP-induced β-sheet formation, reversed by the addition of PKA after five minutes of incubation (b); the influence of ATP during the first two hours of conformational change and effect of two hours delayed phosphorylation (c); and the inverse experiment with PKA (d).

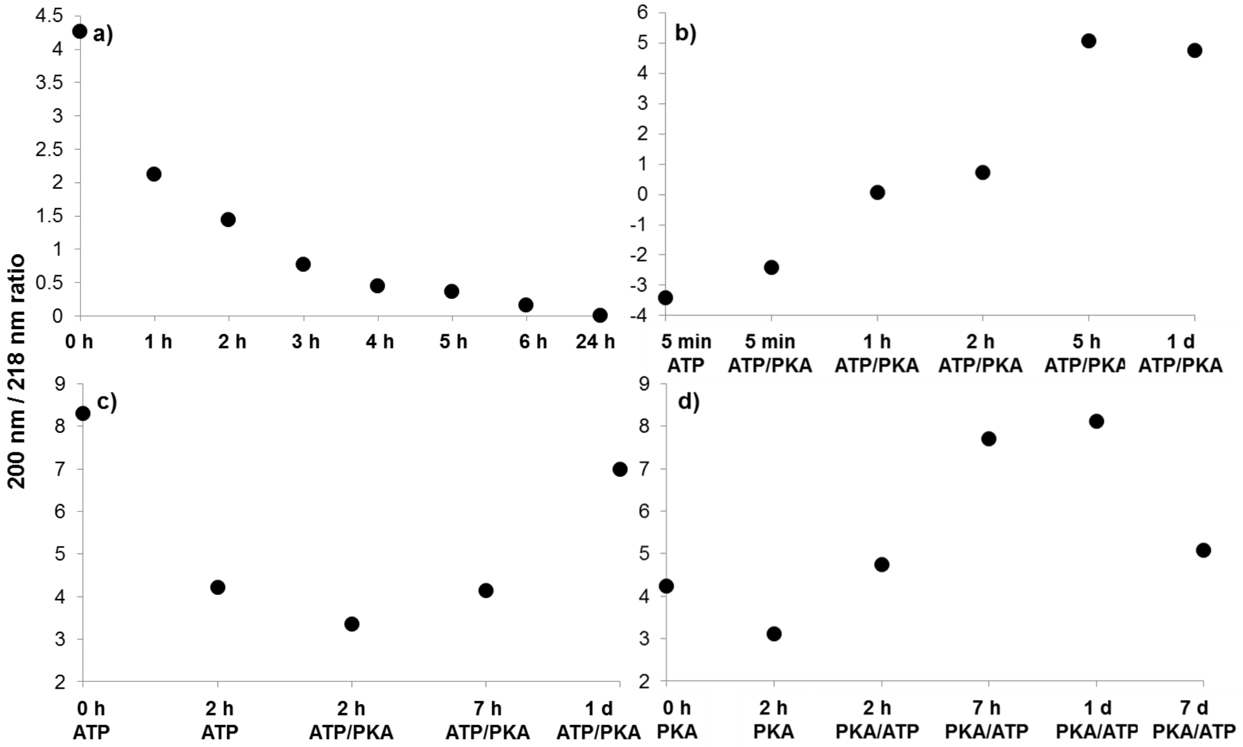

Figure S3: Ratio of 200 nm to 218 nm plotted from CD spectra of enzymatic phosphorylation of 15 µM KFM6 in time-dependent CD-minimum plots. Time-dependent transition from random-coil to β-sheets of plain KFM6 (a); ATP-induced β-sheet formation, reversed by the addition of PKA after five minutes of incubation (b); the influence of ATP during the first two hours of conformational change and effect of two hours delayed phosphorylation (c); and the inverse experiment with PKA (d). The 5 min ATP and 5 min ATP/PKA values of (b) are not divided by 200 nm and plot only 218 nm quantity because 200 nm value was zero.

4. ^{31}P NMR

22 µM of peptide KFM6 was incubated with 200 µM ATP and 5000 U PKA in 50 mM Tris/HCl buffer with 10 mM $MgCl_2$ at pH 7.5 and 27 ± 3 °C. ATP transfers one phosphate during enzymatic phosphorylation to the peptide, leaving an ADP molecule. Compared to ATP, ADP has no β-phosphate with respect to the electronic properties, while the α-phosphate remains constant. The integral ratio from β-phosphate to α-phosphate was followed to determine the reaction ratio to ADP, which is linear proportional to the phosphorylation ratio of KFM6. The pointed data were accumulated for 25 min each. The resulting linear fit curve implies no significant change in the integral ratio during the examined time dimension.

^{31}P NMR (202 MHz, PKA reaction buffer, 27 ± 3 °C, ppm) δ = 6.31 (d, γ-phosphate), 11.45 (d, α-phosphate), 19.66 (dd, β-phosphate).

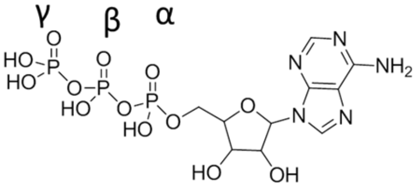

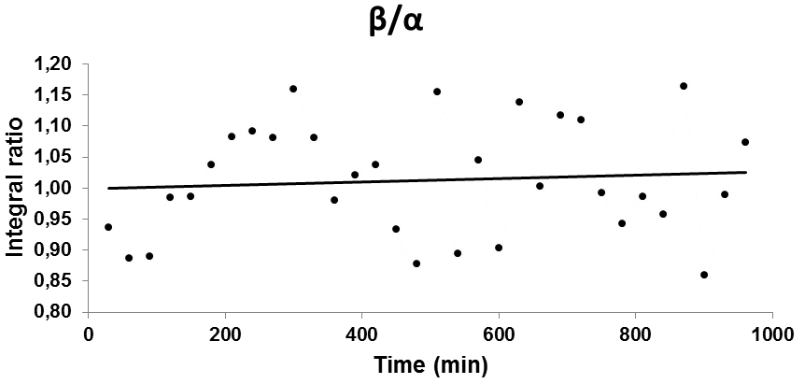

Figure S4: Structure of ATP (left) and ^{31}P NMR experiment with the integrated ration of β- to α-phosphate as a reaction control for the kinetic of the enzymatic phosphorylation (right).

5. TANGO

The computer algorithm TANGO was used to predict the propensities for α-helices, β-sheets or β-aggregates like amyloids [S1-S3]. The calculations were performed for 293 K, at pH 7.5 and an ionic strength of 0.02. The resulting propensities are plotted over the amino acid sequence of KFM6.

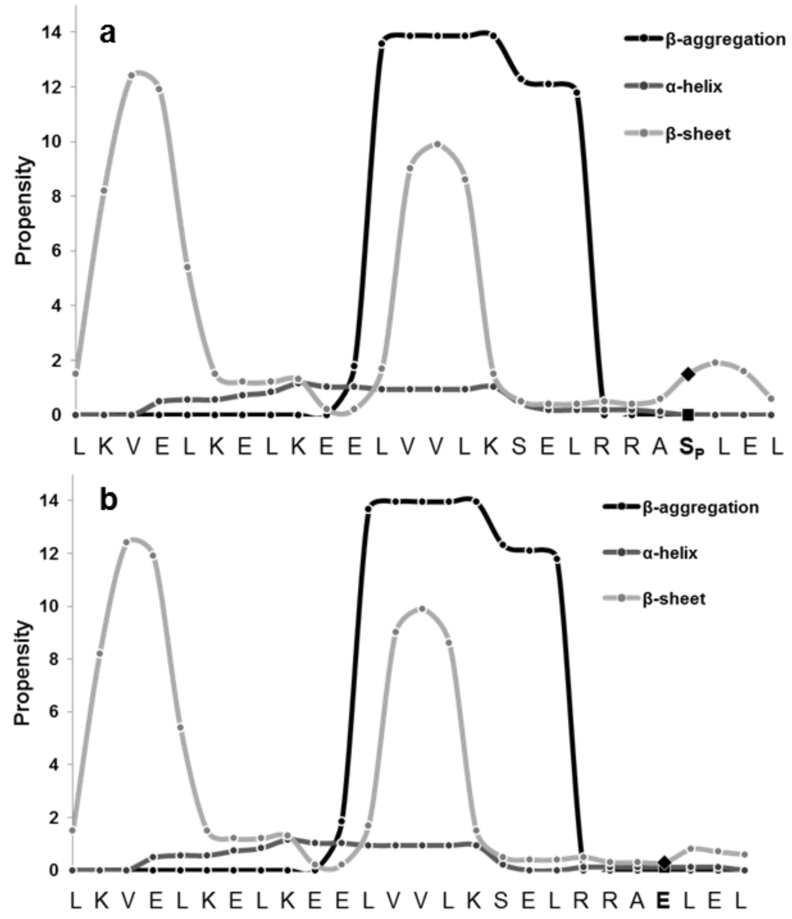

Figure S5: TANGO calculation for the secondary structure propensities of KFM6 with a) serine or b) glutamic acid in position 23.

References

S1. Rousseau, F., Schymkowitz, J., Serrano, L., *Curr. Opin. Struct. Biol.* **2006**, *16*, 118-126.

S2. Fernandez-Escamilla, A.-M., Rousseau, F., Schymkowitz, J., Serrano, L., *Nat. Biotech.* **2004**, *22*, 1302-1306.

S3. Linding, R., Schymkowitz, J., Rousseau, F., Diella, F., Serrano, L., *J. Mol. Biol.* **2004**, *342*, 345-353.
